# Coffee and Cocoa By-Products as Valuable Sources of Bioactive Compounds: The Influence of Ethanol on Extraction

**DOI:** 10.3390/antiox14010042

**Published:** 2025-01-01

**Authors:** Blanca Martínez-Inda, Nerea Jiménez-Moreno, Irene Esparza, Carmen Ancín-Azpilicueta

**Affiliations:** 1 Analytical Chemistry Group, Science Department, Public University of Navarre, 31006 Pamplona, Spain; blanca.martinez@unavarra.es (B.M.-I.); nerea.jimenez@unavarra.es (N.J.-M.); irene.esparza@unavarra.es (I.E.); 2 Institute for Advanced Materials (INAMAT^2^), Universidad Pública de Navarra, 31006 Pamplona, Spain

**Keywords:** cocoa bean shell, spent coffee grounds, solid–liquid extraction, GRAS solvents, phenolic compounds, melanoidins, antioxidant activity

## Abstract

Cocoa and coffee are two of the world’s most important crops. Therefore, their by-products are generated in large quantities. This work proposes a simple method for the valorization of these residues by obtaining phenolic compounds and melanoidins by solid–liquid extraction using different hydroalcoholic solutions as extracting solvents (0, 25, 50, 75, 100% ethanol). Extracts of both by-products presented the highest antioxidant capacity and total phenolic and melanoidin content when using 50–75% ethanol in the solvent. Among all the extracts, those obtained from spent coffee grounds at 75% ethanol showed the highest concentrations of total phenolic compounds (13.5 ± 1.3 mmol gallic acid equivalents/g dry matter) and melanoidins (244.4 ± 20.1 mg/g dry matter). Moreover, the sun protection factor values of the coffee extracts obtained with 50 and 75% of ethanol as extraction solvent (7.8 ± 0.9 and 8.5 ± 0.7, respectively) showed their potential for use in the cosmetic sector. The most important phenolic compounds identified in the coffee by-products extracts were phenolic acids, and most of them were found in higher concentration in extracts obtained with lower percentages of ethanol (0–25%). Protocatechuic acid was the most abundant phenolic in cocoa extracts, with concentrations ranging from 18.49 ± 2.29 to 235.35 ± 5.55 µg/g dry matter, followed by 4-hydroxybenzoic acid, (-)-epicatechin and (+)-catechin. Esculetin was found in both coffee and cocoa extracts, which had not been reported to date in these residues. In summary, the use of 75% ethanol as an extraction solvent seems a good strategy to obtain extracts rich in phenolic compounds from food by-products rich in melanoidins, such as coffee and cocoa by-products. The high antioxidant potential of these extracts makes them of great interest for the cosmetic and nutraceutical industries.

## 1. Introduction

The production of agrifood waste has been, and continues to be, a major environmental problem. Only in 2021, more than 58 million tons of waste (fresh mass) were generated in the European Union, of which around 12 million tons came from processing and manufacturing, and more than 5 million tons came from the production sector [1]. In this context, the new trends in circular economy and biowaste management are focused on waste valorization to obtain value added products that can be of interest for different applications [2]. An example of a by-product generated in great quantities that can be used to obtain compounds of interest is cocoa. *Theobroma cacao* is a tropical tree, belonging to the Malvaceae family, native to the tropical forests of the upper Amazon region [3]. According to the International Cocoa Organization (ICCO), about fifty million people depend on cocoa for their livelihood. In 2021, the annual production of cocoa beans was approximately 4.2 million metric tons, with the main producers being Ivory Coast, Ghana, Indonesia, Brazil, Nigeria, Cameroon, Ecuador, and Colombia [4]. Of the total cocoa yield, only 10% corresponds to the bean, and the rest consist of non-profitable residues [5,6]. Cocoa bean shell, pulp, and husks are the main by-products of the cocoa industry and a source of bioactive compounds. These wastes are not fully utilized and are dumped in the field without any treatment, generating environmental issues [3]. For this reason, it is of great importance to explore alternative uses of cocoa pod husks and bean shells. These materials are mainly composed of fiber, carbohydrates, lignin, proteins, and minerals [7,8]. They are also rich in polyphenols, methylxanthines, and phytosterols, which can be extracted and subsequently used in the field of pharmaceutics, medicine, nutraceuticals, and functional foods [7,9].

Another important source of by-products that generates environmental and economic issues and that can be valorized to obtain high value-added products is coffee. Coffee is the most consumed functional beverage worldwide. Different beneficial health effects are attributed to its consumption, such as anti-obesity, anti-inflammatory, antidiabetic, and antihypertensive properties, all of them related to its strong antioxidant potential [10]. Coffee belongs to the Coffea genus of the botanical family Rubiaceae, with more than 100 species currently known. However, only the arabica and robusta varieties, derived, respectively, from *Coffea arabica* and *Coffea canephora*, are commercially produced and distributed worldwide [11]. Different coffee by-products are produced at different stages of the production chain, from crop residues to spent coffee grounds. The latter are residues obtained from coffee prepared with hot water or steam. Approximately 6 million tons of these residues are produced each year [12] and represent a major pollution hazard if discharged into the environment. These residues contain many valuable substances, but when introduced directly into the soil they can result toxic to microorganisms and plants [13]. Although the World Health Organization considers coffee as “non-nutritive dietary component”, it contains numerous bioactive compounds [14]. Currently, there are several strategies for the reuse of coffee grounds as biofuel [15], and also in the cosmetic industry [16]. In fact, a wide variety of value-added products can be obtained from this coffee by-products: biofuels, bio-sugar, bio-oil, bioactive compounds, enzymes, organic acids, biopolymers, carotenoids, bio-sorbents, antioxidants, and other bio-compounds [17]. In view of the high level of bioactive molecules contained in spent coffee grounds, many scientific studies have focused on finding valorization strategies for this coffee waste as natural source of antioxidants of interest to the nutraceutical and food industries [18]. Therefore, it is possible to obtain extracts rich in bioactive compounds, such as caffeine, chlorogenic acid, caffeic acid, cafestol, and kahweol, from spent coffee grounds [17,19].

In addition to the aforementioned compounds, the two vegetable matrices have melanoidins in their composition as a result of non-enzymatic browning reactions that take place during the roasting processes. Melanoidins are heterogeneous nitrogen-containing brown pigments with very different molecular weight, which are produced by the Maillard reaction in food [20]. Several studies have shown that melanoidins possess antioxidant potential, as well as other relevant biological properties, such as prebiotic, antihypertensive, antiadhesive, and metal ion chelating activity [21,22]. The presence of phenolic compounds, especially acids, linked to the skeleton of the melanoidin structure, has been associated with these biological activities [21].

In view of all of this, the present work aims to find a simple strategy for the extraction of antioxidant compounds from coffee and cocoa by-products using GRAS solvents (water and ethanol), which can be used for different industrial applications. Ethanol is a bio-based solvent that can be manufactured from plant biomass, which is its main renewable source [23]. This solvent is characterized by its low toxicity, renewability, and biodegradability [24]. In contrast, methanol, which is another widely used solvent in the valorization of agrifood waste, is considered less appropriate than ethanol in the context of green chemistry due to its toxicity and health risk [25]. Previous studies conducted by this research group showed that, among the different factors to be considered during process optimization (solvent composition, temperature, incubation time, solid–liquid ratio), the concentration of ethanol of the extraction solvent is the one that most influences the extraction of phenolic compounds [26]. Considering that coffee and cocoa are by-products generated in large amounts, it is very important to continue research in lines that allow for their recovery, as there are still knowledge gaps to be filled. The novelty of this work is that, in the same study, antioxidants are extracted from two food wastes that are generated in a very important way in the world and that have a common characteristic—their high content of melanoidins, which are analyzed. These compounds can bind to the phenolic compounds present in these plant matrices and, thus, influence the extraction process. Therefore, the aim of this work was to study the influence of different hydroalcoholic mixtures on the extraction of phenolic compounds in food by-products with a significant content of melanoidins, such as cocoa bean shell and spent coffee grounds.

## 2. Materials and Methods

### 2.1. Samples and Extraction Conditions

In the present work, two plant by-products were used: cocoa bean shell (CBS), which is the outer shell of cocoa bean, and spent coffee grounds (SCG) from capsules elaborated with a mixture of robusta (*Coffea canephora*) and arabica (*Coffea arabica*) coffee. The coffee waste was collected from household residues and the CBS was bought in Chocolates Comes (Sueca, Valencia, Spain). Both by-products were dried until a constant weight was reached in an oven at 25–30 °C. After that, they were milled using a coffee grinder (Moulinex, Ecully, France) and sieved to obtain a homogeneous 500 µm powder.

The extracts from the above by-products were obtained in all cases by solid–liquid extraction. The selection of the extraction conditions was based on previous studies conducted by the research group [26], in which we concluded that the influence of solid–liquid ratio and temperature were of low relevance in comparison with the solvent composition. In this work, the solid–liquid ratio of 1:100 and a temperature of 40 °C have been used for all the experiments, and the influence of five different hydroalcoholic solutions were studied (0%, 25%, 50%, 75%, and 100% ethanol). First, 100 mL of the water–ethanol mixture was added to 1.00 g of each sample. The extractions were performed in a stove (Ing Climas, Barcelona, Spain) for 24 h under orbital agitation (250 rpm). Subsequently, the samples were centrifuged at 8000 rpm for 15 min (Sorvall ST 8 centrifuge, Thermo Scientific, Waltham, MA, USA) and filtered. The solvent was removed from the resulting supernatant with a rotary evaporator (R-210, Büchi, Barcelona, Spain) at 40 °C. Then, the resulting extracts were resuspended in a small volume of water, frozen, and finally lyophilized. To calculate the extraction yield, the weight of the dry extract obtained after freeze-drying was subtracted from the initial weight of dry matter used for the extraction. The difference was then divided by the initial weight of dry matter and multiplied by 100. Three different extractions were made for each different extraction solvent. The lyophilized extracts were kept refrigerated at 4 °C until analysis.

### 2.2. Antioxidant Capacity

The antioxidant capacity of the extracts was determined by three different methods: ABTS, DPPH, and FRAP. Prior to analysis, 5 mg of each extract was dissolved in 1 mL of methanol and, when necessary, further diluted with the same solvent at the appropriate ratio to fall within the linear range of each analytical method. The ABTS [2,2′-azinobis (3-ethylbenzothiazoline-6-sulphonic acid)] method was based on the method described by Re et al. [27]. The ABTS^• +^ radical cation was obtained by mixing a 7 mM ABTS solution with potassium persulfate 2.45 mM and allowing the mixture to react for 16 h. The absorbance of the ABTS^• +^ was measured at 734 nm and adjusted to 0.70 ± 0.02. The DPPH (2,2-diphenyl-1-pycrilhydracyl) radical scavenging assay used was based on the method proposed by Brand-Williams et al. [28]. A standard solution of DPPH was prepared by weighing 25 mg of DPPH in 100 mL of methanol. The DPPH radical was then diluted to an absorbance of 0.90 ± 0.05 at 517 nm. The third method used was FRAP (Ferric Reducing Antioxidant Power) in which the method developed by Benzie and Strain [29] was taken as a reference. FRAP reagent was prepared by mixing 300 mM acetate buffer (pH 3.6), 10 mM TPTZ (2,4,6-tripyridyl-s-triazine) solution and 30 mM FeCl_3_·6H_2_O solution (10:1:1). The absorbance was measured at 595 nm. A UV/Vis spectrophotometer (Jenway 7315, Stafford, UK) was used to determine the absorbance in all cases. For sample preparation, between 5.0 and 5.3 mg of cocoa or coffee extract was dissolved in 1 mL of methanol. Then, the mixture was sonicated (Ultrasons-HD, Selecta, Barcelona, Spain) for 20 min and filtered through 0.45 µm PTFE syringe filters. For the three methods, Trolox was used as reference standard for the calibration curve and results were expressed as mmol Trolox/g dry matter. The concentrations of the calibration standards ranged from 0.052 to 2.065 mM in ABTS, 0.052 to 0.620 mM in DPPH and 0.052 to 1.187 mM in FRAP. The coefficient of determination R2 was above 0.998 in all cases. In the case of the ABTS method, 2.97 mL of the reagent was added to 30 µL of the Trolox standard or sample, while in FRAP and DPPH methods, 2.85 mL of the reagent was mixed with 150 µL of standard or sample. After 30 min in darkness, the samples were measured at the corresponding wavelength in triplicate.

### 2.3. Total Polyphenol Content

Total polyphenol content (TPC) of the cocoa and coffee extracts was analyzed by the Folin–Ciocalteu method proposed by Singleton et al. [30]. The method consists of adding 7.9 mL of deionized water and 0.5 mL of the Folin–Ciocalteu reagent to 100 µL of sample or standard. After two minutes, 1.5 mL of 20% sodium carbonate in water was mixed and the solution was left in darkness for two hours. The absorbance of the samples was measured at 765 nm in the same UV/Vis spectrophotometer. The standard used for the calibration curve was gallic acid at concentrations between 0.203 and 4.569 mM. In all cases, R^2^ > 0.998. The results were expressed in mmol of gallic acid equivalents/g of dry matter. Each cocoa/coffee extract was analyzed in triplicate.

### 2.4. Melanoidins

For the quantification of melanoidins, extract samples were prepared in triplicate at a concentration of 0.2 mg dry extract/mL in methanol. Melanoidin concentration was determined by measuring the absorbance at 405 nm in 1 cm path length cuvettes and using a specific extinction coefficient (Kmix) of 0.7 L/cm·g, according to Bekedam et al. [31] for the calculation of the concentration through the Lambert–Beer law as follows (1):

K_mix_ (L·cm^−1^·g^−1^) = absorbance/concentration (g·L^−1^) (1)

All the extracts were analyzed in triplicate with the same UV/Vis spectrophotometer.

### 2.5. Sun Protection Factor (SPF)

The sun protection factor (SPF) was determined by an in vitro method developed by Mansur et al. [32] through a simple Formula (2):

(2) $$\mathrm{SPF}=\mathrm{CF}\cdot \sum_{290}^{320}\left[EE\left(\lambda \right)\cdot I\left(\lambda \right)\cdot abs\left(\lambda \right)\right]$$

CF: Correction factor (10);

EE(λ): erythemal efficiency spectrum;

I(λ): solar simulator intensity spectrum;

abs(λ): absorbance value of the sample at the defined wavelength (intervals of 5 nm at UVB range 290–320 nm).

The values of the product EE(λ)·I(λ) are the normalized values proposed by Sayre et al. [33]. The samples were measured in the same spectrophotometer and in triplicate at 0.2 mg of dry extract/mL of methanol.

### 2.6. Identification of Phenolic Compounds by HPLC-MS/MS

A first qualitative determination of the individual phenolic compounds was made on a HPLC-ESI-QTRAP-MS/MS system consisting of an Exion LC AC system connected to a QTRAP 4500 (Sciex, Framingham, MA, USA), and equipped with a heated electrospray ionization source (ESI). The chromatographic separation was conducted on a Kinetex Biphenyl column (5 µm, 2.1 × 150 mm, Phenomenex, Torrance, CA, USA), attached to a security guard precolumn (Phenomenex, Torrance, CA, USA). The mobile phases were composed of 0.1% formic acid in water (solvent A) and 0.1% formic acid in methanol (solvent B). The solvents gradient was: 0 min, 5% B; 2 min, 5% B; 8 min, 40% B; 16 min, 50% B; 24 min, 55% B; 26 min, 100% B; 28 min, 100% B; 30 min, 5% B; and 35 min, 5% B. The flow rate was 0.3 mL/min, and the injection volume was 10 µL. Finally, the column oven temperature was set at 40 °C, while the temperature of the autosampler was set at 15 °C. To analyze the samples, 1 mg of dry extract was dissolved in 1 mL of methanol. The solutions were sonicated (Ultrasons-HD, Selecta, Barcelona, Spain) for 20 min and then filtered through 0.22 µm PTFE syringe filters. All the solvents were LC-MS grade from Carlo Erba (Val de Reuil Cedex, France).

For HPLC-MS/MS analysis, positive electrospray ionization (ESI+) or negative electrospray ionization (ESI-) with scheduled multiple reaction monitoring (sMRM) mode were used to identify precursor and fragment ions of each target analyte. The ESI operation parameters were set as follows: source temperature 450 °C, curtain gas 35 psig, ion source gas 1 (GS1) 60 psig, ion source gas 2 (GS2) 60 psig, and collision gas set to high. The entrance potential (EP) and collision cell exit potential (CXP) were both −10 V in negative mode and 10 V in positive mode. The ion capillary voltage was set to −4500 V in negative mode and 4500 V in positive mode. The optimized collision energy (CE) and declustering potential (DP) for each analyte, together with the three (in some cases two) transitions with the highest signal-to-noise ratios are reported in Table S1 (see Supplementary Material).

### 2.7. Quantification of Phenolic Compounds by HPLC-DAD-FLD

Once identified, the main phenolic compounds were quantified on an Arc HPLC System (Waters, Milford, MA, USA) equipped with a quaternary solvent manager-R (QSM-R), a 2998 Photodiode Array Detector and a 2475 Multi λ Fluorescence Detector. The column used was XSelectTM Premier HSS T3 (2.5 µm, 4.6 × 150 mm, Waters, Milford, MA, USA). Data acquisition and processing were performed by Empower 3.7.0 (Waters, Milford, MA, USA). The method was composed of two mobile phases: A (water: acetic acid, 98:2 *v*/*v*) and B (methanol: acetic acid, 98:2 *v*/*v*). The methanol solvent used to prepare mobile phase B was HPLC plus gradient grade from Carlo Erba (Val de Reuil Cedex, France). The acetic acid glacial was obtained from VWR Chemicals BDH (Rue d’Aurion, France) with 99.8% purity. The flow rate was 1 mL/min and gradient was as follows: 0 min, 95% A; 1 min, 95% A; 5 min, 90% A; 10 min, 88% A; 20 min, 87% A; 30 min, 86% A; 45 min, 84% A; 55 min, 70% A; 63.5 min, 68.3% A; 64.4 min, 68.2% A; 66.4 min, 68.2% A; 68.5 min, 67.6% A; 75 min, 60% A; 79 min, 55% A; 87 min, 50% A; 92 min, 40% A; 94 min, 20% A; 104 min, 0% A; 106 min, 0% A; 111 min, 95% A; and 116 min, 95% A. The injection volume was 10 µL, and the column temperature was set at 40 °C. To analyze the samples, 30 mg of dry extract was dissolved in 300 µL of methanol. The solutions were sonicated (Ultrasons-HD, Selecta, Barcelona, Spain) for 20 min and then filtered through 0.45 µm PTFE syringe filters. In this case, three different samples for each ethanol concentration of both cocoa and coffee extracts were analyzed.

Some of the individual phenolic compounds in cocoa and coffee extract were determined by comparing both the UV/Vis spectrum at the characteristic wavelength and the retention time of the corresponding standards. Other compounds were quantified using a fluorescence detector. The maximum absorption or excitation/emission wavelengths of each compound, as well as their retention times, are listed in Table S2 (see Supplementary Material). The quantification of polyphenols was made by calibration curves for each identified compound except for quercetin and quercetin-3-glucoside, which were quantified as quercitrin and quercetin-3-O-galactoside, respectively. The coefficient of determination was higher than 0.997 in all cases. The standards, (+)-catechin, eriodictyol, esculetin, gentisic acid, 4-hydroxybenzoic acid, quercitrin, and quercetin-3-O-galactoside (hyperoside), were from Extrasynthese (Genay, France). Apigenin, epicatechin, gallic acid, neochlorogenic acid, protocatechuic acid, and vanillic acid were from Sigma-Aldrich (Madrid, Spain). Ferulic acid was from Merck (Darmstadt, Germany). Luteolin was from Phytolab (Vestenbergsgreuth, Germany). Chlorogenic acid was from ACROS Organics (New Jersey, USA) and caffeic acid was from Calbiochem (San Diego, CA, USA). In Figures S1 and S2 of Supplementary Materials, chromatograms obtained from extracts from cocoa and coffee byproducts are shown, respectively.

### 2.8. Statistical Analysis

The differences between the variables were determined by the Kruskal–Wallis test with Bonferroni correction. To determine the linear correlation between antioxidant capacity, total polyphenol content, and sun protection factor, Pearson’s correlation coefficient was used. Each type of extraction was performed in triplicate, and each replicate was analyzed once in the case of chromatographic methods (n = 3) and three times in the case of spectrophotometric methods (n = 9). All data were processed with SPSS^®^ statistical software (IBM^®^ SPSS^®^ Statistics for Windows version 28.01.1 Armonk, NY, USA: IBM Corp).

## 3. Results and Discussion

### 3.1. Extraction Yield

The extraction yield (%) of CBS was around 26% when the extraction solvents used were 0, 25, and 50% ethanol. With increasing ethanol concentration, the CBS extraction yield decreased, with the lowest value (5%) being obtained when using only ethanol as extracting medium. However, in the case of SCG, the extraction yield increased with increasing ethanol content in the extracting solvent. The lowest yield was obtained when using water (6.3%), and the highest value was obtained with 75% ethanol (20%). SCG come from domestic coffee waste, so they have been previously extracted with water during coffee brew. This fact could explain the low extraction yield obtained when using high water contents in the extracting medium of SCG. CBS, however, is a residue from the cocoa industry that has not undergone any prior extraction. The yield results are given in Figure S3 of the Supplementary Material.

### 3.2. Antioxidant Activity, Melanoidin, and Total Phenolic Content

**Figure 1:**
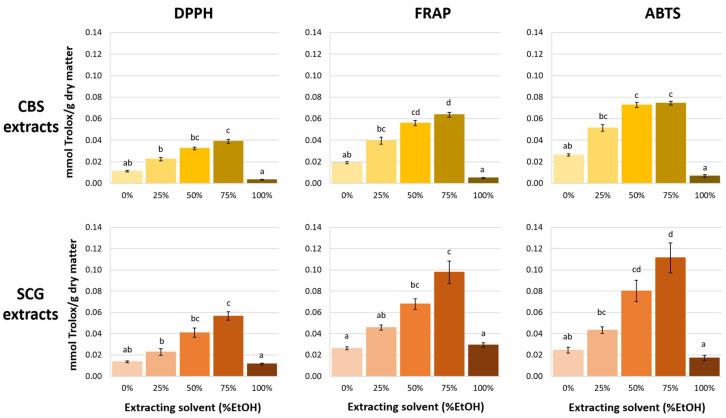
Antioxidant activity (mmol Trolox/g dry matter) of cocoa bean shell (CBS) and spent coffee grounds (SCG) extracts measured by three methods (DPPH, FRAP and ABTS). Different letters indicate significant differences according to the extracting solvent (*p* < 0.01).

The results of antioxidant activity of the CBS and SCG extracts can be found in Figure 1. As it can be seen, values obtained using the DPPH method provided lower Trolox-equivalent values for all the extracts than the FRAP and ABTS methods. This difference is probably due to the steric accessibility problems that the DPPH radical can present [34] and that hinder its reaction with large molecules, such as melanoidins, which are present in both type of by-products [35]. On the other hand, the antioxidant capacity profile of the extracts obtained with increasing solvent ethanol concentration was similar regardless of the method used in the determination (Figure 1). Both CBS and SCG extracts showed the highest antioxidant capacity when 50 or 75% ethanol were used as extraction solvents, while the lowest antioxidant activity was found in extracts obtained with 100% ethanol. Several authors [26,36,37,38,39] have observed that the antioxidant capacity of extracts from different vegetable matrices (including CBS and SCG) is greater when using hydroalcoholic mixtures than when using pure solvents. Therefore, according to these results, it seems that ethanol–water mixtures can dissolve a broader range of antioxidant compounds than using pure water or ethanol. Furthermore, the use of mixtures of solvents can induce swelling of the plant matrix, which is very rich in carbohydrate polymers, facilitating the penetration of the solvent and increasing the diffusion rate [40,41].

Comparing both SCG and CBS extracts, it can be seen that SCG extracts showed higher antioxidant activity than CBS extracts (Figure 1) when the percentage of ethanol used in the extraction was 50% or higher. Since the antioxidant capacity of these food matrices is generally attributed to the presence of both phenolic compounds from plant matrix and melanoidins generated during cocoa or coffee roasting from carbohydrates and amino groups/proteins [42], the melanoidin and total phenolic content in both extracts were determined by UV/Vis spectrophotometry. Table 1 shows the content of melanoidins and phenolic compounds in CBS and SCG extracts. Within each type of extract, the highest levels of melanoidins and phenolic compounds were found when 50% and 75% ethanol were used as extraction solvents, which coincides with the most antioxidant extracts (Figure 1). Therefore, both melanoidins and total phenolic content were highly correlated with the antioxidant capacity of the extracts, with Pearson’s correlation values from 0.894 to 0.993 (see Pearson’s correlations in Table S3 in Supplementary Material).

Comparing CBS and SCG extracts, it was found that the concentration of melanoidins was similar in both extracts when water was used as extraction solvent. However, when the percentage of ethanol was 25% or higher, the SCG extracts presented higher values of melanoidins than the CBS extracts. The greatest differences were found in the extracts obtained with 50% and 75% ethanol, which agrees with the results of antioxidant activity.

In the case of total phenolic content (TPC), SCG extracts showed similar values to that found by other authors in spent coffee grounds extracts [43,44]. In fact, Panusa et al. [45], who analyzed SCG extracts also from spent capsules, found 12.6 mg of gallic acid/g of dry weight in extracts obtained using 60% ethanol as extraction solvent, a value very similar to those found in the present study. Likewise, the phenolic content of CBS extracts was found in the range between 7.1 and 11.7 mg of gallic acid/g of dry matter reported by Grillo et al. [46] in cocoa bean shell extracts. When comparing TPC in SCG and CBS extracts, higher values were found in SCG extracts than in CBS ones in all cases, even though SCG had undergone a previous extraction (coffee brew) while the CBS were a raw waste. As in the case of melanoidins, the greatest differences between CBS and SCG extracts were found in those obtained with 50 and 75% of ethanol, which also coincides with the results of antioxidant capacity.

The good coherence found between the results of melanoidin content, total phenolic content, and antioxidant activity in both types of extracts suggest that the three parameters are closely related. Therefore, it is confirmed that both melanoidins and phenolic compounds influence on the antioxidant capacity of the extracts, but also that there exists a good correlation between the content of both types of compounds (see Pearson’s correlations in Table S3 in Supplementary Material). This good correlation could be explained by considering how melanoidins are formed during food processing. During the roasting process of coffee and cocoa, phenolic compounds can be incorporated into melanoidin structures [47,48]. Chlorogenic and other hydroxycinnamic acids have been found linked to coffee melanoidins [49,50], and flavanols such as catechin or epicatechin have been detected bound to cocoa melanoidins [51]. The Folin–Ciocalteu reagent, in addition to reacting with the free phenolic compounds in the extract, could be reacting with the melanoidin-bound phenolics, which are much more abundant than the free ones [21]. Moreover, it has been reported that about 50% of the antioxidant activity of melanoidins is due to these melanoidin-bound phenolics [42,52]. Therefore, the higher content of melanoidins found in SCG extracts could also be responsible for the higher total polyphenol content and antioxidant activity observed in these extracts compared to CBS extracts.

### 3.3. Sun Protection Factor (SPF)

**Figure 2:**
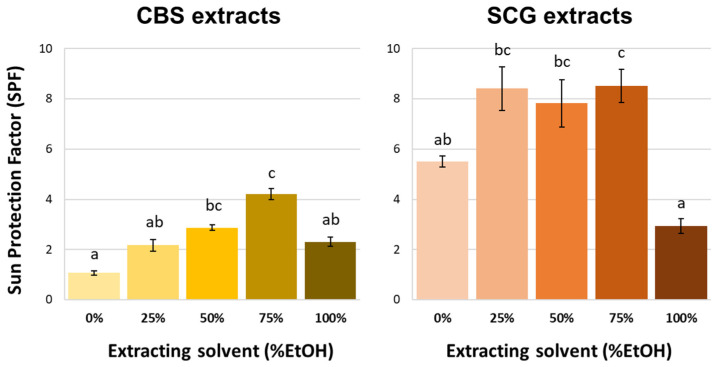
Sun Protection Factor (SPF) at a concentration of 0.2 mg/mL of cocoa bean shell (CBS) and spent coffee grounds (SCG) extracts. Different letters indicate significant difference using Kruskal–Wallis test with Bonferroni correction at 0.01 level of significance (*p* < 0.01).

The sun protection factor (SPF) of the different extracts is summarized in Figure 2. As can be seen, a fairly similar profile was obtained in CBS and SCG extracts, although SPF values were higher in SCG than in CBS extracts. Page et al. [53] characterized SCG ethanolic extracts obtained from six different spent espresso capsules. These authors found that the sun protection factor of all the extracts, measured at the same concentration than the one used in the present work (0.2 mg/mL), were between 2.3 and 2.8, values very close to that found in the present work in the SCG extract obtained with 100% ethanol (2.9 ± 0.3). SPF values in cosmetic products are in the range of 6 to 50 [54]. Therefore, the SCG extracts obtained in this work with 25 to 75% ethanol could be used in the cosmetic sector at the concentration of this study (0.2 g/mL), while those from CBS should be used in higher concentration.

Considering the influence of the solvent used for extraction in the SPF values, it can be concluded that, as observed for the other spectrophotometric parameters, solvents with 50 and 75% ethanol gave rise to extracts with the highest SPF values. However, SPF values were not as closely related to antioxidant capacity as the content of melanoidins and phenolic compounds, because Pearson’s correlations were found between 0.675 (SPF-ABTS) and 0.759 (SPF-DPPH) for CBS extracts and between 0.693 (SPF-FRAP) and 0.727 (SPF-ABTS) for SCG extracts. In both types of extracts, the best correlations found for SPF values were with TPC values, which agrees with previous studies performed by this research group for different type of extracts from vegetable sources and means that the total phenolic content is the determining factor in the SPF of the extracts [55].

### 3.4. Qualitative Determination of Phenolic Compounds in the Extracts

**Figure 3:**
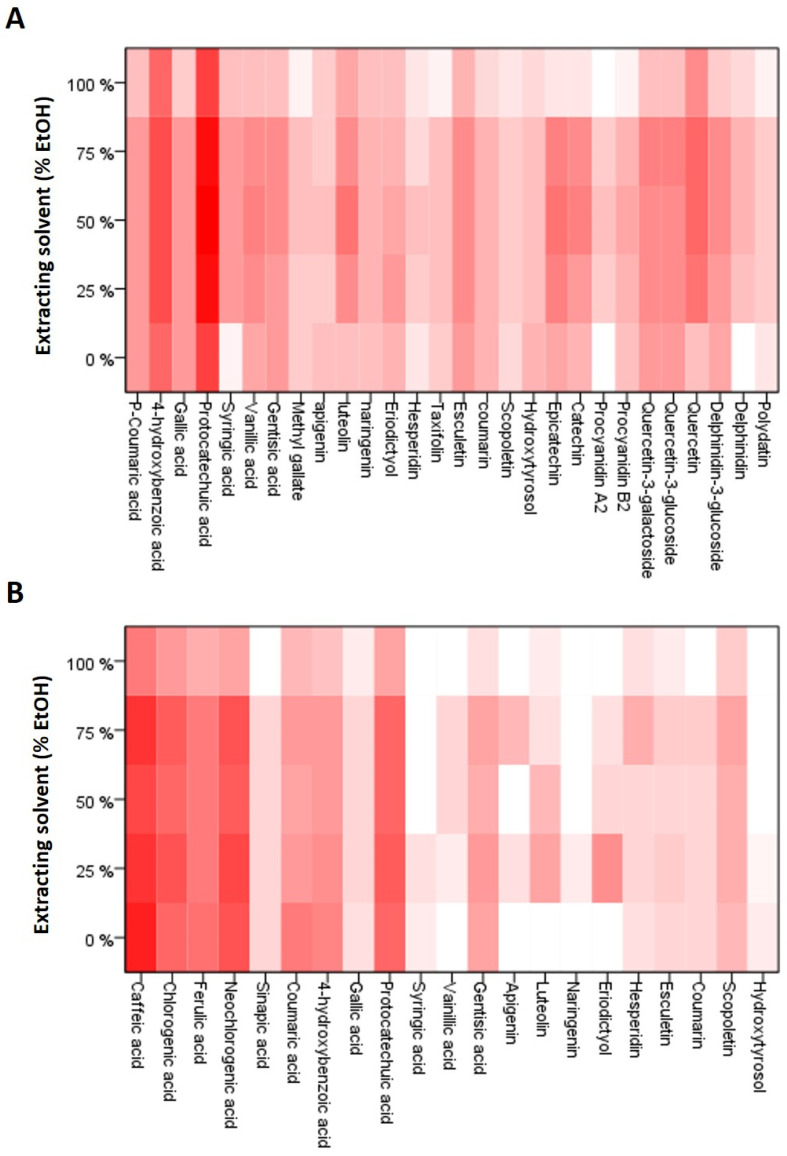
Heatmap of the phenolic compounds identified by HPLC-MS/MS using the peak areas relative to mg of dry matter in CBS (**A**) and SCG (**B**) extracts. The more intense the red color, the greater the signal intensity of the corresponding phenolic compound.

Figure 3 shows the heatmaps designed from the signal intensities obtained for each of the compounds identified in the HPLC-MS/MS analyses of all the extracts. In CBS extracts, eight phenolic acids were identified: seven hydroxybenzoic acids (4-hydroxybenzoic, gallic, protocatechuic, syringic, vanillic, gentisic acid, and methyl gallate, which is a gallic acid derivative) and p-coumaric acid, which was the only hydroxycinnamic acid found in these extracts. In SCG extracts, apart from the hydroxybenzoic acids identified in CBS extracts, six hydroxycinnamic acids (caffeic, chlorogenic, neochlorogenic, ferulic, sinapic, and *p*-coumaric acids) were found. As can be seen in Figure 3B, phenolic acids were the most important phenolic compounds in SCG extracts. The signals obtained for flavonoids, coumarins, and hydroxytyrosol were weak compared to those of these acids. Other works [56,57] have also pointed out the abundance of phenolic acids in the composition of extracts obtained from SCG. In fact, the most important phenolic acids in coffee beans and, therefore, the most discussed in the literature are caffeic acid and its derivatives such as caffeoylquinic acids, commonly called chlorogenic acids [58]. In the case of CBS extracts (Figure 3A), protocatechuic acid was the compound with the highest signal intensity, followed by 4-hydroxybenzoic acid and flavonoids such as quercetin, epicatechin, catechin, and luteolin. Catechin, epicatechin, procyanidin B2, quercetin, and protocatechuic acid have also been described as the major compounds in this plant species [59].

The influence of the percentage of ethanol in the solvent on the extraction profile was different in the two plant matrices studied (Figure 3). In CBS extracts, in general, a greater extraction of phenolic compounds was observed when mixtures of ethanol and water were used as extraction solvent than when pure ethanol or water was used. However, in SCG extracts, the strongest signals were found for the extracts obtained with higher percentages of water. This fact is surprising because the coffee waste used in these extractions had already been previously extracted with water when preparing the coffee. However, the contact time between CBS and water during coffee preparation is very low compared to the 24 h of extraction used in this study.

### 3.5. Quantitative Determination of Phenolic Compounds in the Extracts by HPLC-DAD-FLD

Table 2 includes the concentration of phenolic compounds in CBS extracts. As it can be seen, the most abundant compounds were protocatechuic acid, 4-hydroxybenzoic acid, catechin, epicatechin, and quercetin, with protocatechuic acid being the major phenolic in all of them, regardless of the composition of the extracting solvent. Other authors [60,61,62] have also described protocatechuic acid as one of the most abundant phenolic compounds in CBS extracts. In fact, the concentrations found in this work for protocatechuic acid, 4-hydroxybenzoic acid, catechin, and epicatechin are in the same order of the concentrations found by Rebollo-Hernanz et al. [62] for cocoa bean shell extracts. However, other works [5,46,63] that have studied the phenolic composition of this type of cocoa residue have reported that the main compounds were flavanols, both monomeric and oligomeric. This lack of coherence between different studies may be due to the fact that the phenolic composition in cocoa products varies significantly depending on several factors, but especially on the manufacturing processes [63,64]. This is because after harvesting, cocoa beans undergo fermentation, drying, and roasting to promote both the recovery of cocoa nibs and the development of the cocoa flavor and, during some of these processes, the flavanol content in cocoa products decrease dramatically [63]. Fermentation of cocoa beans can reduce more than 90% of the initial flavanol concentration [65]. Furthermore, flavanols are thermolabile compounds as we found in a previous work on the stability of some phenolic compounds [66]; therefore, roasting results in further flavanol losses as have been reported by other authors [67]. On the other hand, it is important to note that catechins and epicatechins can not only be found free in cocoa but also bound to melanoidins, giving rise to insoluble bound phenolic compounds [21,35], which cannot be detected by chromatographic techniques without prior hydrolysis. Therefore, loss of flavanols and their binding to melanoidins that occur during cocoa processing, and other factors that affect polyphenol content such as geographical origin, variety, plant genotype, and the harvest season [5] could explain the great variability of phenolic content of cocoa products found by different researchers. In this regard, Rojo-Poveda et al. [68] studied the variations in phenolic composition for CBS extracts of different origins and cultivars, finding very wide ranges of concentrations of the different compounds: 11.89–214.01 mg/kg of CBS for protocatechuic acid; 12.72–180.37 mg/kg for catechin; 4.47–748.79 mg/kg for epicatechin; and 1.07–22.37 mg/kg for quercetin. As can be seen in Table 2, the contents of all these compounds found in the different CBS extracts analyzed in the present study were within these concentration ranges.

On the other hand, the esculetin content found in this study for CBS extracts was also remarkable (Table 2). This coumarin is widely distributed in the plant kingdom but, until now and to the best of our knowledge, its presence has not been reported in cocoa by-products. Esculetin has been gaining a lot of interest in recent years due to its chemotherapeutic activity against several types of cancer such as breast, colon, liver, pancreas, and lung cancer, among others [69,70]. Moreover, this coumarin also presents antidiabetic action [70], anti-inflammatory activity, and protects against cardiovascular diseases [71]. Therefore, the finding of esculetin in CBS extracts is important, and its content in commonly consumed cocoa-derived products such as chocolate should be studied.

With the only exception of eriodictyol, the concentration of all the detected and quantified phenolic compounds in CBS extracts increased as the percentage of ethanol in the solvent increased up to 75% ethanol. However, when 100% of ethanol was used as the extraction solvent, the concentration of the phenolic compounds decreased. In the case of eriodictyol, its concentration was very similar and low in all extracts (Table 2). The extracts with the highest content of phenolic compounds were those obtained with 50 and 75% ethanol as extracting solvent, which is consistent with the qualitative results and also with the results of antioxidant capacity and total polyphenol content previously presented (Figure 1 and Table 1).

Regarding SCG extracts, the most abundant phenolic compounds in all extracts were hydroxycinnamic acids, mainly caffeic acid and chlorogenic acids or CGA (Table 3). The term CGA designates a family of esters formed between certain *trans*-hydroxycinnamic acids and (-)-quinic acid and include *p*-coumaroylquinic, feruloylquinic, caffeoylquinic, and dicaffeoylquinic acids [72]. This group of compounds has been reported as the major phenolic compounds in SCG extracts [43,56,57]. The extracts analyzed in the present work showed a higher content of caffeic acid than that of the caffeoylquinic acid isomers, probably due to the previous processing of green coffee beans. During roasting of coffee beans, part of the CGA is isomerized, part is transformed into quinolactones due to dehydration and formation of an intramolecular bond, and part is hydrolyzed releasing the corresponding hydroxycinnamates such as caffeic or ferulic acids [73,74]. Furthermore, the aforementioned isomerization would explain the higher content of neochlorogenic acid (3-caffeoylquinic acid, 3-CQA) compared to chlorogenic acid (5-caffeoylquinic acid, 5-CQA) found in the SCG extracts here analyzed (Table 3). According to the study by Farah et al. [73], during roasting, the levels of chlorogenic acid decreased substantially, while the levels of neochlorogenic acid increased to almost double their original values.

Regarding the influence of the ethanol content in the extracting solvent, although high values of different compounds were found for extracts obtained with 0 to 75% ethanol in the solvent, most of the phenolic compounds were better extracted when using low ethanol levels (0–25% ethanol, Table 3). Several authors [75,76,77] have observed that, regardless of the extraction method used (solid–liquid, Soxhlet, microwave-assisted extraction), the extraction of polyphenols in SCG was higher when using pure water or hydroalcoholic mixtures with alcohol contents below 25%. However, the results of the total content of phenolic compounds obtained by the Folin–Ciocalteu method in the SCG extracts showed higher values when using 50 or 75% ethanol in the solvent. Recently, Kitchen and Williamson [78] have suggested that the determination of phenolic compound content by the Folin–Ciocalteu method in plant-based heat-treated foods produces significant overestimations of this value due to the presence of melanoidins. Therefore, the higher content of total phenolic compounds found in this study for SCG extracts obtained with 50 or 75% ethanol might be due to the greater influence of melanoidins in these extracts and not to a higher presence of free phenolic compounds. On the other hand, the contribution of dicaffeoylquinic acids and 1,5-γ-quinolactones in the extracts obtained with a higher presence of ethanol in the solvent should not be ruled out. These compounds, which are found in a prominent way in coffee beans [73], are less soluble in water than mono-caffeoylquinic acids, which are highly hydrophilic [79] and, therefore, will be better extracted with higher percentages of ethanol.

It should be noted that the extraction profile of protocatechuic acid was different in the two plant matrices studied in this work. While in the SCG extractions the content of this acid was maximum when using pure water, in the CBS extractions higher contents were obtained when using 50 or 75% ethanol. Likely, the different chemical nature of both plant matrices has considerably influenced the extraction of this compound, since it influences the solubility of phenolic compounds [80]. In the literature, different extraction behaviors have been found for the same compound depending on the product from which it is extracted. For example, some authors [26,81] have reported that gallic acid, which is a hydroxybenzoic acid like protocatechuic acid, is better extracted from grape stems or damson plums with pure water than with pure ethanol, while other authors [82] found higher contents of this acid when extractions were performed with 99% ethanol.

Consumer demand for natural products focuses the interest of cosmetic, food, and nutraceutical industries on natural antioxidants. Before a natural antioxidant reaches the market, a complete development is necessary from extraction and characterization to industrial scale-up. Thus, this work focuses on the first phase of this development, demonstrating that it is possible to obtain an extract with antioxidant and photoprotective properties, using a simple and scalable GRAS method. These extracts are of interest for different sectors such as, for example, food preservatives, additives with a dual function in cosmetics (antioxidant and sunscreen), and for the development of smart coatings and nutraceuticals. In the future, safety, efficacy, and scalability studies should be carried out to advance the development of products based on CBS or SCG extracts.

## 4. Conclusions

In this work, the combined use of two sustainable and safe solvents, such as water and ethanol, allowed for the obtainment of extracts with high contents of phenolic compounds from both cocoa bean shells and spent coffee grounds. Considering the antioxidant capacity, melanoidin content, and phenolic composition, the best percentage of ethanol for the extraction from cocoa by-products was 50–75%, while for spent coffee ground extracts, the most effective solvents contained between 25% and 75% ethanol. Taking into consideration the extraction yields, it can be concluded that the best percentages of ethanol in the extraction solvent were 50% for cocoa by-products and 75% for coffee by-products. Most of the compounds found in spent coffee ground extracts were phenolic acids, with caffeic acid being the major one. Phenolic acids were also found in cocoa shell extracts, with protocatechuic acid being the predominant one. However, in this case, considerable concentration of flavanols, such as (+)-catechin and (-)-epicatechin, and flavonols, such as quercetin, were also found. It is also worth mentioning the identification, for the first time, of esculetin in the extracts from both by-products. The high concentration of phenolic compounds and melanoidins found in the extracts together with their high antioxidant potential and their sun protection factor demonstrate that these extracts are of potential interest for use in both the cosmetic and food sectors. In this regard, it has been demonstrated that concentrations of 0.2 mg/mL of CBS extract are enough to obtain SPF values higher than 6, showing their potential as additives for sunscreens in the field of cosmetics.

In some plant matrices, phenolic compounds can be bound to proteins, polysaccharides or, as in the case of cocoa and coffee, to melanoidins, and this fact can condition both their release depending on the extraction conditions and their characterization and quantification by different methods. In this work, the influence of ethanol on the extraction of bioactives from two different vegetal matrices rich in melanoidins has been studied and it has been found that the extracts that showed the highest antioxidant capacity in both cases were those obtained with 75% ethanol in the extraction solvent. Therefore, this ethanolic content seems to be the most suitable for extracting antioxidants from food by-products with a high content of melanoidins.

## Figures and Tables

**Table 1:** Melanoidin and total phenolic content of cocoa bean shell (CBS) and spent coffee grounds (SCG).

	Solvent (% Ethanol)	CBS Extracts	SCG Extracts
Melanoidin content (mg/g dry matter)	0	26.28 ± 4.79 ^a^	22.25 ± 1.66 ^a^
	25	43.40 ± 3.93 ^ab^	72.10 ± 8.15 ^ab^
	50	100.03 ± 13.46 ^b^	181.97 ± 11.52 ^b^
	75	117.47 ± 3.75 ^bc^	244.35 ± 20.10 ^bc^
	100	12.52 ± 2.36 ^a^	47.84 ± 0.57 ^a^
Total phenolic content (mg gallic acid/g dry matter)	0	1.21 ± 0.18 ^a^	3.36 ± 0.26 ^a^
	25	3.22 ± 0.31 ^ab^	6.17 ± 0.61 ^ab^
	50	6.04 ± 0.40 ^b^	10.02 ± 0.70 ^b^
	75	9.15 ± 0.49 ^b^	13.52 ± 1.26 ^b^
	100	1.13 ± 0.13 ^a^	3.52 ± 0.25 ^a^

Different letters indicate significant differences by ethanol concentration in the solvent (*p* < 0.01) using the Kruskal–Wallis test with Bonferroni correction.

**Table 2:** Concentration of phenolic compounds (µg/g dry matter) in cocoa bean shell extracts (CBS) obtained by using different percentages of ethanol in the extraction solvent.

Phenolic Compounds	% Ethanol				
	0%	25%	50%	75%	100%
Vanillic acid **	<0.20 ^a^	6.26 ± 0.27 ^ab^	8.35 ± 0.11 ^ab^	9.28 ± 0.04 ^b^	3.28 ± 0.24 ^ab^
Gallic acid **	0.38 ± 0.11 ^a^	3.45 ± 0.02 ^ab^	5.35 ± 1.06 ^ab^	9.95 ± 0.29 ^b^	3.39 ± 0.22 ^ab^
Protocatechuic acid *	18.49 ± 2.29 ^a^	171.14 ± 12.80 ^ab^	234.89 ± 4.01 ^b^	235.35 ± 5.55 ^b^	59.15 ± 4.71 ^a^
4-Hydroxybenzoic acid *	4.48 ± 0.50 ^a^	15.99 ± 1.00 ^ab^	21.43 ± 0.26 ^b^	21.62 ± 0.52 ^b^	9.09 ± 0.70 ^ab^
(+)-Catechin **	0.98 ± 0.61 ^a^	6.10 ± 4.74 ^ab^	25.39 ± 1.58 ^b^	8.68 ± 0.47 ^ab^	7.45 ± 0.79 ^ab^
(-)-Epicatechin **	2.06 ± 1.57 ^a^	8.32 ± 6.80 ^ab^	41.11 ± 3.29 ^b^	13.25 ± 0.79 ^ab^	11.68 ± 1.33 ^ab^
Quercetin ^¥^ **	0.26 ± 0.10 ^a^	4.72 ± 0.85 ^ab^	14.00 ± 0.93 ^ab^	16.97 ± 1.74 ^b^	2.21 ± 0.56 ^ab^
Quercetin-3-galactoside *	nd ^a^	nd ^a^	0.73 ± 0.02 ^b^	0.87 ± 0.07 ^b^	0.06 ± 0.01 ^ab^
Quercetin-3-glucoside ^§^ **	0.45 ± 0.05 ^a^	0.66 ± 0.15 ^ab^	1.20 ± 0.12 ^ab^	1.34 ± 0.06 ^b^	0.51 ± 0.08 ^ab^
Apigenin *	nd ^a^	<0.12 ^ab^	0.19 ± 0.01 ^ab^	0.20 ± 0.01 ^b^	0.08 ± 0.01 ^ab^
Luteolin *	0.11 ± 0.02 ^a^	0.61 ± 0.03 ^ab^	1.52 ± 0.20 ^b^	1.42 ± 0.01 ^b^	0.33 ± 0.05 ^a^
Eriodictyol *	nd ^a^	<0.08 ^a^	<0.08 ^a^	<0.06 ^a^	0.06 ± 0.00 ^a^
Esculetin **	1.23 ± 0.14 ^a^	3.11 ± 0.21 ^ab^	4.31 ± 0.09 ^ab^	4.65 ± 0.07 ^b^	1.53 ± 0.12 ^ab^

nd: not detected; ^¥^ expressed as quercitrin; ^§^ expressed as quercetin-3-galactoside. Each value represents the means ± SD of three replicates. Different letters on the same row indicate significant differences using the Kruskal–Wallis test with Bonferroni correction (* *p* < 0.05, ** *p* < 0.01).

**Table 3:** Concentration of phenolic compounds (µg/g dry matter) in spent coffee grounds (SCG) obtained by using different percentages of ethanol in the extraction solvent.

Phenolic Compounds	% Ethanol				
	0%	25%	50%	75%	100%
Vanillic acid *	nd ^a^	2.11 ± 0.68 ^b^	1.31 ± 0.17 ^ab^	1.07 ± 0.27 ^ab^	0.63 ± 0.21 ^ab^
Gallic acid	0.39 ± 0.01 ^a^	0.29 ± 0.15 ^a^	0.58 ± 0.13 ^a^	0.55 ± 0.18 ^a^	0.13 ± 0.06 ^a^
Protocatechuic acid *	20.50 ± 5.69 ^b^	18.76 ± 3.24 ^ab^	13.12 ± 1.39 ^ab^	12.44 ± 4.29 ^ab^	3.80 ± 1.86 ^a^
Gentisic acid **	2.69 ± 0.60 ^ab^	8.57 ± 3.78 ^b^	3.20 ± 0.23 ^ab^	2.43 ± 0.59 ^ab^	0.28 ± 0.15 ^a^
4-Hydroxybenzoic acid *	2.07 ± 0.52 ^b^	2.27 ± 1.37 ^ab^	1.48 ± 0.09 ^ab^	1.21 ± 0.39 ^ab^	0.42 ± 0.20 ^a^
Caffeic acid *	358.08 ± 36.59 ^ab^	429.55 ± 22.11 ^b^	321.54 ± 28.19 ^ab^	342.77 ± 83.43 ^ab^	107.41 ± 37.42 ^a^
Chlorogenic acid *	20.33 ± 3.66 ^ab^	61.04 ± 11.87 ^b^	43.39 ± 3.07 ^ab^	49.29 ± 12.01 ^ab^	8.12 ± 2.63 ^a^
Neochlorogenic acid **	67.32 ± 9.86 ^ab^	104.72 ± 16.01 ^b^	85.00 ± 6.46 ^ab^	95.88 ± 27.29 ^ab^	10.07 ± 2.97 ^a^
Ferulic acid **	18.65 ± 0.23 ^b^	12.78 ± 0.06 ^ab^	11.34 ± 2.28 ^ab^	10.29 ± 2.82 ^ab^	3.19 ± 1.11 ^a^
Apigenin **	<0.03 ^a^	0.04 ± 0.00 ^a^	<0.07 ^ab^	<0.09 ^b^	<0.08 ^ab^
Esculetin	0.08 ± 0.01 ^a^	0.08 ± 0.01 ^a^	<0. 01 ^a^	0.11 ± 0.02 ^a^	0.07 ± 0.00 ^a^

nd: not detected. Each value represents the means ± SD of three replicates. Different letters on the same row indicate significant differences using Kruskal–Wallis test with Bonferroni correction (* *p* < 0.05, ** *p* < 0.01).

## Data Availability

The raw data supporting the conclusions of this article will be made available by the authors on request.

## Supplementary Materials

The following supporting information can be downloaded at: https://www.mdpi.com/article/10.3390/antiox14010042/s1, Figure S1: Chromatograms of cacao 75% ethanol extract displayed at 294 (red) and 350 (black) nm; Figure S2: Chromatograms of coffee 0% ethanol extract displayed at 294; Figure S3: Extraction yield (%) of cocoa bean shell (CBS) and spent coffee ground (SCG) using different ethanol levels in the extraction solvent; Table S1: Optimized collision energy (CE) and declustering potential (DP) for each analyte, together with the transitions with the highest signal-to-noise ratios; Table S2: Retention times (Rt) and maximum absorption or excitation/emission (ex/em) wavelengths for each analyte; Table S3: Pearson’s correlation matrix (*p* < 0.01) among antioxidant activity methods (DPPH, FRAP and ABTS), melanoidins content, total phenolic content (TPC), and sun protection factor (SPF) in cocoa bean shell (CBS) and spent coffee grounds (SCG) extracts.
