# Supplementary material for: Coffee and Cocoa By-Products as Valuable Sources of Bioactive Compounds: The Influence of Ethanol on Extraction
Source: Antioxidants (Basel). 2025 Jan 1;14(1):42. doi: 10.3390/antiox14010042 (PMC11762683; doi:10.3390/antiox14010042)
Supplement: Supplementary file 1 [file antioxidants-14-00042-s001.zip › antioxidants-3391645-supplementary-2.pdf]

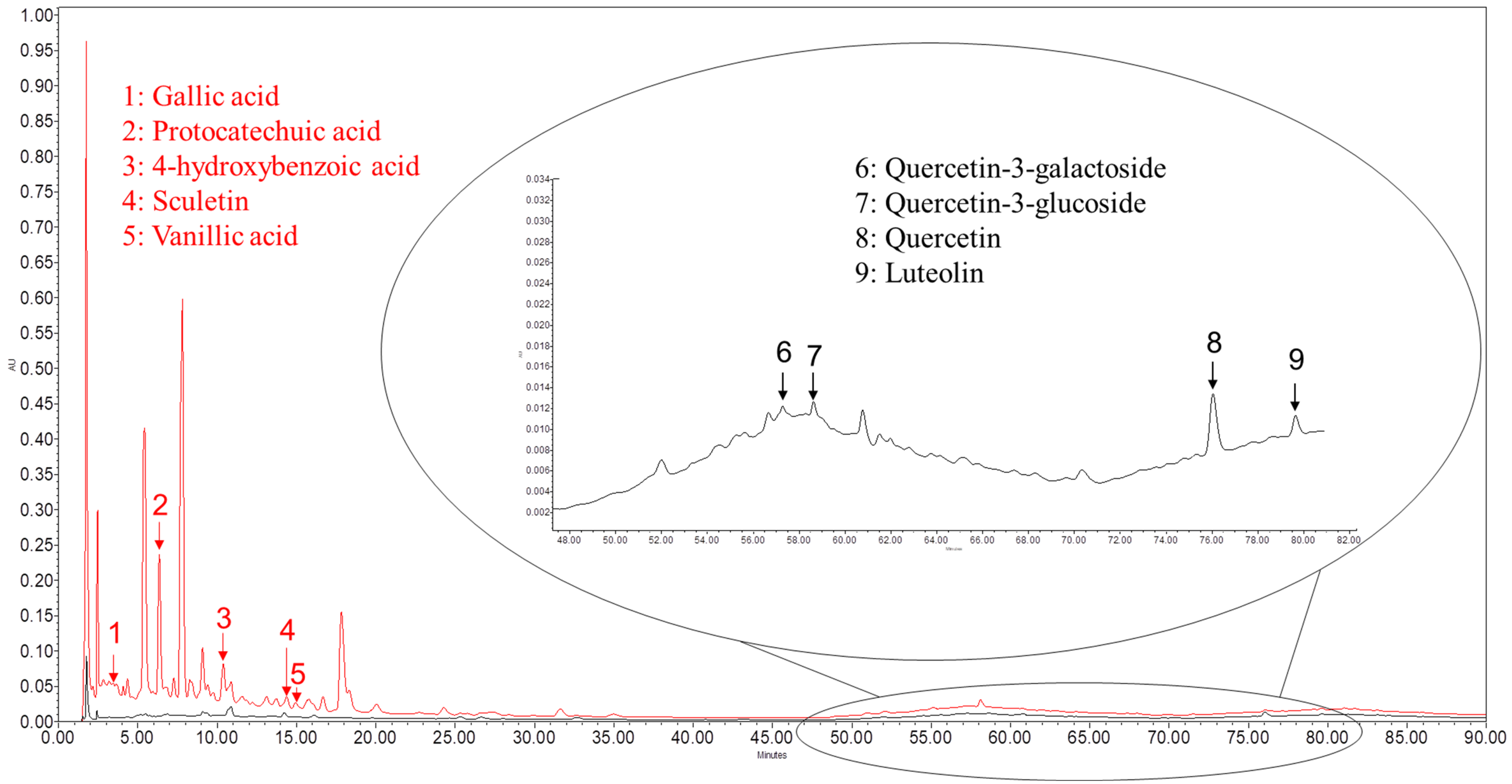

Figure S1. Chromatograms of cacao 75% ethanol extract displayed at 294 (red) and 350 (black) nm.

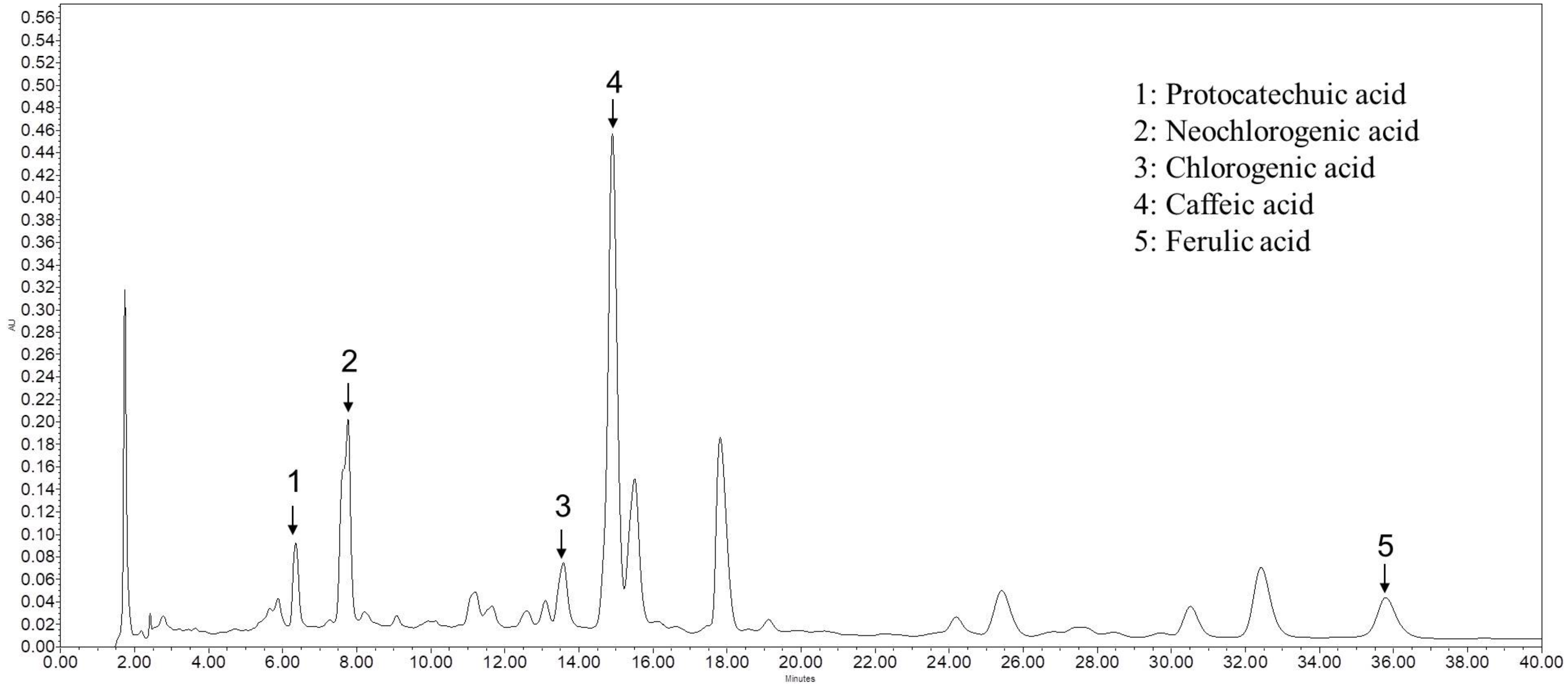

Figure S2. Chromatograms of coffee 0% ethanol extract displayed at 294.

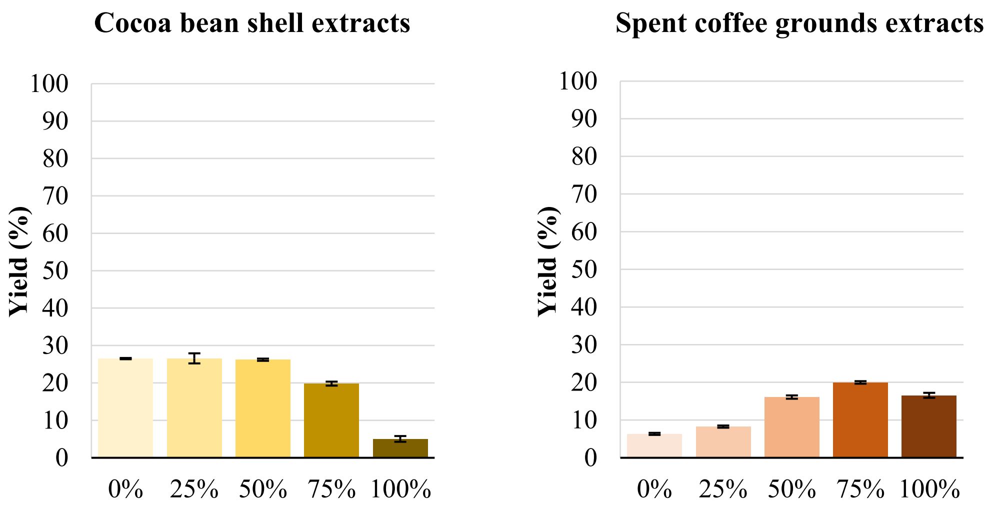

Figure S3. Extraction yield (%) of cocoa bean shell (CBS) and spent coffee ground (SCG) using different ethanol levels in the extraction solvent

Table S1. Optimized collision energy (CE) and declustering potential (DP) for each analyte, together with the transitions with the highest signal-to-noise ratios.

Analyte	Rt (min)	Parent Ion (*m/z*)	Product Ions (Quantifier/qualifier/confirming) (*m/z*)	Declustering Potential (V)	Collision Energy (V)	λ (nm)
HYDROXYCINNAMIC ACIDS & DERIVATIVES						
caffeic acid	8.9	179.0	135 / 134 / 89	-20	-20 / -30 / -40	324
p-coumaric acid	10.3	163.0	119 / 93 / 117.5	-40	-20 / -40 / -50	308
chlorogenic acid	9.9	353.0	191 / 85 / 127	-20	-40 / -50 / -50	326
ferulic acid	11.5	193.0	134 / 178 / 149	-60	-30 / -20 / -20	322
caftaric acid	6.2	311.0	149 / 135 / 179	-20	-20 / -40 / -20	329
neochlorogenic acid	6.7	353.0	191 / 135 / 179	-20	-40 / -50 / -40	324
cinnamic acid	17.2	147.0	103 / 77	-60	-20 / -30	277
chicoric acid	10.7	472.5	311 / 149 / 179	-20	-20 / -30 / -40	329
trans-coutaric acid	8.3	295.0	163 / 119 / 149	-20	-30 / -30 -30	313
sinapic acid	12.2	223.0	208 / 164 / 149	-20	-20 / -20 / -30	323
verbascoside	11.4	623.0	161 / 133 / 315	-40	-40 / -100 / -40	332
HYDROXYBENZOIC ACIDS & DERIVATIVES						
gallic acid	2.1	169.0	125 / 97 / 80	-40	-20 / -30 / -40	271
vanillic acid	10.3	167.0	152 / 124 / 108.5	-40	-20 / -20 / -20	290
ellagic acid	12.1	301.0	145 / 185 / 229	-60	-50 / -40 / -40	367
protocatechuic acid	4.18	153.0	109 / 108 / 123	-60	-20 / -40 / -20	280
syringic acid	10.2	197.0	182 / 123 / 95	-40	-20 / -30 / -40	274
gentisic acid	6.16	153.0	108 / 81	-40	-30 / -20	327
4-hydroxybenzoic acid	7.12	137.0	93 / 65 / 75	-20	-20 / -40 / -40	255
methyl gallate	8.2	183.0	124 / 78 / 168	-80	-30 / -40 / -20	273
ethyl gallate	10.2	197.0	124 / 169 / 78	-60	-30 / -20 / -50	273
FLAVONOIDS						
FLAVANOLS						
(+)-catechin	8.1	289.0	245 / 123 / 109	-80	-20 / -40 / -20	280
(-)-epicatechin	9.6	289.0	245 / 123 / 109	-80	-20 / -40 / -50	279
(-)-epigallocatechin	7.8	305.0	125 / 179 / 138	-20	-30 / -20 / -40	233
epicatechin 3-O-gallate	10.6	441.0	169 / 125 / 289	-80	-30 / -50 /-30	323

(-)-epigallocatechin gallate	9.7	457.0	169 / 125 / 305	-60	-50 / -30 / -30	275
catechin 3-O-gallate	10.7	441.0	169 / 125 / 289	-80	-30 / -60 / -30	278
procyanidin A2	11.1	577.0	286.5 / 424.5 / 137.5	60	40 / 20 / 80	278
procyanidin B1	9.4	578.0	289 / 409 / 275	20	30 /30 / 30	277
procyanidin B2	9.4	579.0	291 / 409 / 426.5	60	20 / 30 / 20	279
theaflavin	18.4	563.0	241 / 269 / 137	-40	-50 / -50 / -60	456
FLAVONOLS						
quercetin	15.7	301.0	151 / 179 / 121	-80	-30 / -30 /-50	371
quercetin 3-glucoside	11.6	463.0	300 / 271 / 179	-20	-50 / -40 /-40	356
quercetin 3-glucuronide	11.5	477.0	301 / 179 / 151	-60	-40 / -50 / -40	356
quercetin 3-galactoside	11.6	463.0	300 / 271 / 255	-60	-40 / -50 / -50	356
quercetin 3,4’-di-O-glucoside	10.5	625.0	463 / 301 / 179	-40	-30 / -50 / -50	343
rutin	11.4	609.0	300 / 271 / 151	-40	-50 / -40 / -50	323
kaempferol	18.6	285.0	185 / 159 / 151	-60	-40 / -50 / -40	366
myricetin	12.7	317.0	179 / 151 / 137	-40	-330 / -30 / -40	373
isorhamnetin	20.8	315.0	300 / 151 / 107	-80	-30 / -40 / -50	369
quercitrin	12.8	447.0	300 / 271 / 151	-40	-30 / -50 / -50	349
kaempferide	27.7	299.0	284 / 151 / 107	-20	-30 / -40 / -50	364
morin	13.7	301.0	151 / 125 /107	-60	-30 / -30 - /40	354
fisetin	13.3	285.0	135 / 121 / 91	-100	-30 / -30 / -40	361
FLAVONES						
apigenin	19.6	269.0	117 / 151 / 107	-80	-38 / -30 / -40	332
luteolin	16.8	285.0	133 / 151 / 175	-80	-40 / -50 / -30	253
apigenin 7-glucoside	14.1	431.0	268 / 211 / 239	-140	-50 / -80 / -60	337
luteolin 7-glucoside	12.1	447.0	285 / 133 / 151	-140	-40 / -80 / -60	350
tangeretin	28.5	373.0	183 / 299 / 327	60	60 / 50 / 50	323
diosmin	16.5	607.0	299 / 284 / 256	-80	-30 / -60 / -100	346
FLAVANONES						
taxifolin	10.8	303.0	285 / 125 / 151	-80	-20 / -30 / -30	285
naringin	14.4	579.0	271 / 151 / 175	-80	-40 / -50 / -40	283
naringenin	18.8	271.0	151 / 119 / 107	-40	-30 / -30 / -30	289
eriodictyol	15.4	287.0	151 / 135 / 107	-80	-20 / -40 / -30	286
hesperetin	22.2	301.0	164 / 136 / 151.5	-40	-40 / -40 / 50	287
hesperidin	16.5	609.0	301 / 164 / 286	-80	-50 / -60 / -50	287
narirutin	13.9	579.0	271 / 151 / 119	-40	-30 / -60 - /80	334

ISOFLAVONOIDS						
daidzein	16.5	253.0	208 / 132 / 223	-80	-40 / -50 / -50	301
genistein	19.3	269.0	133 / 159 /63	-80	-40 / -40 / -60	260
ANTHOCYANINS						
malvidin	10.2	331.0	315 / 287 / 241.5	30	40 / 50 / 50	540
cyanidin	10.7	287.0	241 / 137 / 213	30	40 / 40 / 40	530
delphinidin	15.7	303.0	229 / 257 / 202	40	50 / 50 / 40	535
peonidin	21.6	301.0	287 / 259	40	40 / 40	532
pelargonidin	12.8	271.0	121 / 198 / 93	80	50 / 40 / 60	517
petunidin	11.3	317.0	246 / 203 / 274	60	50 / 50 / 40	539
malvidin 3-O-glucoside	10.3	493.0	331 / 316 / 287.5	20	30 / 80 / 80	534
cyanidin 3-glucoside	9.6	449.0	287 / 213 / 241	80	40 / 80 / 80	520
delphinidin 3-O-glucoside	11.6	465.0	303 / 229 / 257	40	40 / 70 / 50	526
cyanidin 3-O-rutinoside	9.8	595.0	287 / 449 / 213	60	50 / 30 / 50	522
peonidin 3-O-glucoside	10.3	462.0	201 / 229 / 257	100	80 / 80 / 60	519
pelargonidin 3-O-glucoside	10.1	433.0	272 / 141 /169	40	40 / 80 / 80	505
petunidin 3-O-glucoside	9.7	479.0	317 / 303 / 217	20	30 / 60 / 80	530
malvidin 3,5-O-diglucoside	8.5	655.0	331 / 492 / 315	40	50 / 30 / 80	530
cyanidin 3,5-O-diglucoside	8.5	611.0	287 / 449 / 213	20	50 / 30 / 120	515
DIHYDROCHALCONES						
phloretin	17.0	273	123 / 81	-40	-30 / -40	287
phloridzin	12.6	435	273 / 167 / 123	-60	-20 / -40 / -50	349
STILBENES						
ε-viniferin	16.6	453	347 / 225 / 198	-80	-40 / -50 / -50	332
trans-resveratrol	14.4	227	143 / 185 / 119	-20	-30 / -20 / -40	307
polydatin	12.7	389	227 / 185 / 143	-80	-30 / -50 / -50	305
piceatannol	12.0	243	201 / 159 / 175	-80	-30 / -30 / -30	323
astrignin	11.0	405	243 / 159 / 201	-40	-30 / -60 / -50	291
LIGNANS						
hinokinin	28.3	353	336 / 96 / 318	40	20 / 40 / 30	287
pinoresinol	19.2	357	151 / 136	-80	-20 / -30	287
secoisolariciresinol	13.9	361	165 / 121 / 179	-80	-30 / -50 / 40	350
magnolin	27.9	417	279 / 327 / 380	20	10 / 20 / 20	277
enterolactone	20.1	297	253 / 107 / 189	-80	-30 / -40 / -30	275

COUMARINS						
coumarin	16.0	147	103 / 91 / 65	40	20 / 40 / 50	268
esculetin	9.7	177	105 / 89 / 133	-60	-30 / -30 / -20	356
scopoletin	12.8	191	176 / 104 / 148	-40	-20 / -30 / -30	345
umbelliferone	11.9	161	133 / 105 / 77	-80	-30 / -30 / -40	323

Table S2. Retention times (Rt) and maximum absorption or excitation/emission (ex/em) wavelengths for each analyte.

Analyte	Rt (min)	DAD	FLD
		λ (nm)	ex/em (nm)
HYDROXYCINNAMIC ACIDS & DERIVATIVES			
caffeic acid	15.0	-	340 / 420
chlorogenic acid	13.6	325	-
ferulic acid	35.9	322	-
neochlorogenic acid	7.8	325	-
HYDROXYBENZOIC ACIDS & DERIVATIVES			
gallic acid	3.5	271	
vanillic acid	15.0	-	290 / 333
protocatechuic acid	6.4	260 / 294	-
gentisic acid	9.9	327	-
4-hydroxybenzoic acid	10.3	255	-
FLAVONOIDS			
FLAVANOLS			
(+)-catechin	10.6	-	290 / 333
(-)-epicatechin	20.4	-	290 / 333
FLAVONOLS			
quercetin	76.0	255 / 364	-
quercetin 3-glucoside	58.7	256 /355	-
quercetin 3-galactoside	57.3	256 / 355	-
quercitrin	65.2	256 / 349	-
FLAVONES			
apigenin	85.5	266 / 339	-
luteolin	79.7	255 / 350	-
FLAVANONES			
eriodictyol	64.6	288	-
COUMARINS			
esculetin	14.3	297 / 345	-

Table S3. Pearson's correlation matrix ($p<0.01$) among antioxidant activity methods (DPPH, FRAP and ABTS), melanoidins content, total phenolic content (TPC) and sun protection factor (SPF) in cocoa bean shell (CBS) and spent coffee grounds (SCG) extracts.

	**Cocoa Bean Shell (CBS) extracts**						**Spent Coffee Grounds (SCG) extracts**					
	**DPPH**	**FRAP**	**ABTS**	**Melan.**	**TPC**	**SPF**	**DPPH**	**FRAP**	**ABTS**	**Melan.**	**TPC**	**SPF**
**DPPH**	1	0.997	0.986	0.959	0.948	0.759	1	0.986	0.988	0.971	0.986	0.693
**FRAP**	0.997	1	0.994	0.948	0.931	0.732	0.986	1	0.982	0.958	0.993	0.695
**ABTS**	0.986	0.994	1	0.931	0.894	0.675	0.988	0.982	1	0.962	0.986	0.727
**Melan.**	0.959	0.948	0.931	1	0.964	0.808	0.971	0.958	0.962	1	0.965	0.611
**TPC**	0.948	0.931	0.894	0.964	1	0.900	0.986	0.993	0.986	0.965	1	0.727
**SPF**	0.759	0.732	0.675	0.808	0.900	1	0.693	0.695	0.727	0.611	0.727	1
